# Brain structural connectivity increases concurrent with functional improvement: Evidence from diffusion tensor MRI in children with cerebral palsy during therapy

**DOI:** 10.1016/j.nicl.2015.01.002

**Published:** 2015-01-09

**Authors:** Zoë A. Englander, Jessica Sun, Laura Case, Mohamad A. Mikati, Joanne Kurtzberg, Allen W. Song

**Affiliations:** a Brain Imaging and Analysis Center, Duke University Medical Center, United States; b Department of Biomedical Engineering, Duke University Medical Center, United States; c Department of Pediatrics, Duke University Medical Center, United States; d The Robertson Cell and Translational Therapy Center, Duke University Medical Center, United States; e Division of Physical Therapy, Duke University Medical Center, United States; f Department of Radiology, Duke University Medical Center, United States

**Keywords:** Cerebral palsy, Diffusion tensor imaging, Structural connectome, GMFM-66

## Abstract

Cerebral Palsy (CP) refers to a heterogeneous group of permanent but non-progressive movement disorders caused by injury to the developing fetal or infant brain (Bax et al., 2005). Because of its serious long-term consequences, effective interventions that can help improve motor function, independence, and quality of life are critically needed. Our ongoing longitudinal clinical trial to treat children with CP is specifically designed to meet this challenge. To maximize the potential for functional improvement, all children in this trial received autologous cord blood transfusions (with order randomized with a placebo administration over 2 years) in conjunction with more standard physical and occupational therapies. As a part of this trial, magnetic resonance imaging (MRI) is used to improve our understanding of how these interventions affect brain development, and to develop biomarkers of treatment efficacy. In this report, diffusion tensor imaging (DTI) and subsequent brain connectome analyses were performed in a subset of children enrolled in the clinical trial (n = 17), who all exhibited positive but varying degrees of functional improvement over the first 2-year period of the study. Strong correlations between increases in white matter (WM) connectivity and functional improvement were demonstrated; however no significant relationships between either of these factors with the age of the child at time of enrollment were identified. Thus, our data indicate that increases in brain connectivity reflect improved functional abilities in children with CP. In future work, this potential biomarker can be used to help differentiate the underlying mechanisms of functional improvement, as well as to identify treatments that can best facilitate functional improvement upon un-blinding of the timing of autologous cord blood transfusions at the completion of this study.

## Highlights

• DTI was used to assess changes in WM connectivity in children with CP.
• Changes in connectivity correlate with changes in functional abilities during therapy.
• Changes in connectivity were diffusely distributed throughout the brain.
• Brain connectivity is a potential biomarker for functional improvement in CP.

## 1 Introduction

Cerebral Palsy (CP) is estimated to affect 3–4 out of 1000 children (Yeargin-Allsopp et al., 2008) and consists of disordered movement, often in conjunction with deficits in sensation, cognition, communication, and behavior (Bax et al., 2005; Aisen et al., 2011). A variety of disturbances in the developing fetal or infant brain may lead to CP, with the resulting neurological deficits correlated with degree and location of damage to brain structure (Accardo et al., 2004). Brain damage in CP often consists of diffuse damage and/or focal lesions in white matter (WM), which are often most severe in periventricular regions (Haynes et al., 2003). While CP is typically diagnosed via neurological assessment, neuroimaging techniques such as *T_2_*-weighted imaging, and more recently diffusion MRI, have been used to characterize WM abnormalities associated with functional deficits in this disorder at a single time point (for a systematic review see Scheck et al. (2012)).

There is extensive literature on neuroimaging studies concerned with functional recovery in brain disorders, (Staudt et al., 2006; Sawaki et al., 2008; Sharma et al., 2009; Pajonk et al., 2010; Bosnell et al., 2011; Johansen-Berg, 2012; Madhavan et al., 2014). Neuroimaging studies specifically in CP have indicated relationships between functional and structural changes within discrete anatomical regions, mostly focusing on sensorimotor regions of interest (ROIs) (Trivedi et al., 2008; Jain et al., 2014). Additionally, initial evaluations of the efficacy of experimental treatments for CP – including autologous stem cell therapy – have been performed (Bae et al., 2012; Lee et al., 2012; Min et al., 2013), also using neuroimaging metrics derived from discrete brain regions to demonstrate structural changes associated with functional improvement.

However, there have been several studies demonstrating damage to WM tracts throughout the brain (Nagae et al., 2007), as well as diffuse connectivity deficits associated with severity of functional impairment (Englander et al., 2013; Pannek et al., 2014) at a single time point. Furthermore, CP is a heterogeneous disorder with multiple causes and clinical manifestations, meaning that the specific structural changes that may underlie improved function are likely to be unique to each patient. These factors indicate that longitudinal studies in CP should explore structural change throughout the brain on an individualized basis, in addition to examining specific changes within the sensorimotor network.

Therefore, in this report we use diffusion tensor imaging (DTI) and whole brain connectome analyses to investigate connectivity changes throughout the brain in relation to functional outcomes in children with CP. To maximize the potential for functional improvement, all children in this trial received autologous cord blood transfusions in conjunction with more standard physical and occupational therapies. The aim of this report was to investigate neuroimaging biomarkers that would reflect diffusely distributed and heterogeneous changes in connectivity in relation to improved functional outcomes following therapy. In future analyses, this biomarker can be used to determine the underlying mechanisms of these functional improvements, potentially helping to identify the treatments that best facilitate better functional outcomes.

## 2 Materials and methods

In this report we used diffusion tensor imaging (DTI) and whole brain connectome analysis to investigate connectivity changes throughout the brain in relation to functional outcomes in 17 children with CP, who all showed positive but varying degrees of functional improvement over the first 2 years of a longitudinal study. We specifically investigated whether brain connectivity changes could serve as a biomarker for improved functional outcomes during therapy in children with CP.

### 2.1 Subjects

Neuroimaging and functional data were analyzed in a subset of children enrolled in our ongoing clinical trial to evaluate the impact of various treatments (including autologous cord blood infusions) for CP. These children had a clinical diagnosis of CP, with either unilateral or bilateral impairment. MRI and functional assessments were scheduled at three time points over a 2-year period, each separated by one year. The children received an autologous cord blood transfusion in either the first or second year, with a placebo administered in the alternate year. The time point at which the experimental treatment was administered was randomized across subjects, and the researchers analyzing the imaging data were blind to the time point at which the treatment was administered. All children had received a transfusion by the time of the final MRI session. Patients underwent neurological testing of motor control, muscle tone and spasticity, overall flexibility and reflexes. Children were sedated for the MRI scans to limit subject discomfort and motion artifacts. Written informed consent was obtained from the parents of each participant, and study related procedures were approved by the Duke University Medical Center Institutional Review Board.

Children were excluded from this report if they had a seizure disorder, brain dysmorphogenesis, or genetic disease. An additional exclusion criterion was significantly abnormal brain anatomy (such as in the case of perinatal stroke) that would prohibit robust image registration or parcellation. 25 subjects had completed the functional and neuroimaging assessments at both time points, however 8 subjects for which an accurate anatomical parcellation could not be achieved were not included in further analyses. These subjects had major anatomical abnormalities due to stroke. Therefore, 17 children (median age = 2.4 years, age range 1.1–5.1 years at time of enrollment) are included in this report. Demographic information for these children is presented in Table 1.

### 2.2 Rehabilitative therapies

In addition to autologous cord blood transfusions, the children in this study received rehabilitation services in their home communities which may have included physical therapy (PT), occupational therapy (OT), developmental therapy, (DT), speech/language therapy (LT), hippotherapy, vision or hearing therapy, and the use of orthotic intervention and adaptive equipment, as are typically included in the comprehensive management of CP. A comprehensive list of therapies is included in Table 2.

### 2.3 Functional outcome measures

The Gross Motor Function Classification System (GMFCS) levels are used to evaluate functional impairment at the time of enrollment. The GMFCS is a five level classification system (Levels I–V) appropriate for the assessment of young children, with distinctions between the levels based on functional limitations and the need for assistive mobility devices (Palisano et al., 1997). Children classified at Level I have the least impaired motor function, whereas children classified at Level V show the most severe functional impairment.

The Gross Motor Function Measure-66 (GMFM-66), the most commonly utilized functional outcome measure in children with CP (Wang and Yang, 2006; Alotaibi et al., 2014), is used to assess changes in functional abilities during treatment in this study. The GMFM-66 includes the assessment of quality of movement in addition to the acquisition of age related isolated skills (Russell, 2002). Children in this report demonstrated GMFM-66 score changes ranging from 2 to 22 points. Here we use a GMFM-66 score change of 10 as a threshold to stratify the subjects into two groups, a moderate improvement group (GMFM-66 score change < 10) and a significant improvement group (GMFM-66 score change > 10). These groups separate subjects based on their levels of functional improvement over the 2-year period. This threshold was chosen based on the distribution of GMFM-66 score changes in the cohort, and it allowed for balanced numbers within each group as well as a highly significant (p = 0.0003) difference in the mean change scores associated with each group. The group of children with GMFM-66 change scores < 10 (n = 9) had a mean GMFM-66 change score of 4.44 ± 1.77, and the group of children with GMFM-66 change scores > 10 (n = 8) had a mean GMFM-66 change score of 15.5 ± 3.61. This group distinction allowed us to assess whether structural characteristics and functional abilities at the time of enrollment have an impact on responsiveness to therapy.

### 2.4 Image acquisition

Diffusion weighted images were acquired on a 3 Tesla GE MR750 scanner (Waukesha, WI) using a 25-direction gradient encoding scheme at b = 1000 s/mm^2^ with 3 non-diffusion-weighted images. An echo time (TE) of 70.5 ms and a repetition time (TR) of 12,000 ms were used. An isotropic resolution of 2 mm^3^ was achieved using a 96 × 96 acquisition matrix in a field of view (FOV) of 192 × 192 mm^2^. *T_1_*-weighted images were obtained with an inversion-prepared 3D fast spoiled-gradient-recalled (FSPGR) pulse sequence with a TE of 2.5 ms, an inversion time (TI) of 450 ms, a TR of 6.5 ms, and a flip angle of 12°, at a 1 mm^3^ isotropic resolution.

### 2.5 Region of interest parcellation

Region of interest (ROI) parcellation was performed by warping the JHU-DTI-MNI “Eve” atlas template (Oishi et al., 2009; Faria et al., 2010; Faria et al., 2011) into each subject's DTI image space via the Advanced Normalization Tools (ANTs) toolkit (Avants et al., 2009), and the parcellation results were visually inspected to confirm anatomical consistency. The individual regions of this atlas that are associated with the brainstem were combined to create a single ROI. The bilateral putamen was removed from the final set as its proximity to the ventricles created inaccurate parcellation for many subjects. As a result, a total of 61 regions were defined for each individual, 30 gray matter regions in each hemisphere, and a single region encompassing the brainstem. The total WM volume of each subject was obtained using FSL FAST (Zhang et al., 2001).

### 2.6 White matter tractography and filtering

After data inspection and rejection (if necessary) for motion artifacts, diffusion tensors were derived from the DTI dataset. Deterministic tractography was performed in the whole brain using the fiber assignment by continuous tracking (FACT) streamline tracking algorithm (Mori et al., 1999; Mori and Zhang, 2006). Streamlines were spline and length filtered; streamlines that were less than 20 mm or longer than 500 mm were removed. With the diffusion and parcellation images in the same image space, streamlines were classified by which ROIs contain their origination and termination points. Streamlines were discarded if they did not start and end in an ROI (Daducci et al., 2012; Englander et al., 2013). Tractography, parcellation, and streamline classification were performed via a standardized pipeline for each patient without regard for the patients' clinical classification.

### 2.7 Brain connectome analysis

In the whole-brain connectome analysis, gray matter ROIs (defined by the parcellation scheme) are defined as “nodes” and the mutual connectivities between pairs of nodes (the volume of voxels containing the streamlines that originate and terminate within a pair of gray matter ROIs) are defined as “edges”. Baseline connectivity was determined by obtaining the sum of the edge values, normalized by the total WM volume, for each child at the time of enrollment. Baseline fractional anisotropy (FA) was obtained by averaging the FA within the voxels that make up the edges at the time of enrollment.

The relationships between changes in connectivity and functional improvements over the first 2-year period of the study were analyzed for all nodes and edges. Specifically, the relationship between connectivity change between any given node and the rest of the brain, and functional improvement was examined. In the edge analysis, the correlation between the mutual connectivity change between each pair of nodes and functional improvement was assessed. Overall mutual connectivity changes were calculated for each child by obtaining the difference in the sum of edge values (normalized by the change in total WM volume, and scaled by the maximum increase in total connectivity across the cohort) throughout the brain and between specific sensorimotor regions. These metrics are referred in this report as total connectivity change and sensorimotor connectivity change. Here the sensorimotor regions are limited to those associated with the primary motor pathway, specifically, the bilateral pre- and post-central gyri, the bilateral thalamus, and the brainstem.

The aim of this whole-brain connectome analysis was to assess the changes in brain connectivity resulting from combined therapy. To visualize these changes in each individual, the increases in connectivity were illustrated in the form of a connectome map, with the thickness of each edge proportional to the magnitude of its connectivity change.

### 2.8 Assessment of relationships between age, brain structure, and functional improvement

Baseline connectivity was compared between children showing moderate functional improvements (GMFM-66 change scores < 10) and children who achieved more pronounced functional improvements (GMFM-66 change scores > 10) using the Mann–Whitney U test. Additionally, baseline FA and mean GMFCS level at the time of enrollment was compared between these groups of children. The relationships between age at time of enrollment and GMFM-66 score change, total and sensorimotor connectivity changes were examined; and correlations between structural and functional measures, as well as age, were performed using the Spearman rank correlation.

## 3 Results

**Fig. 1:**
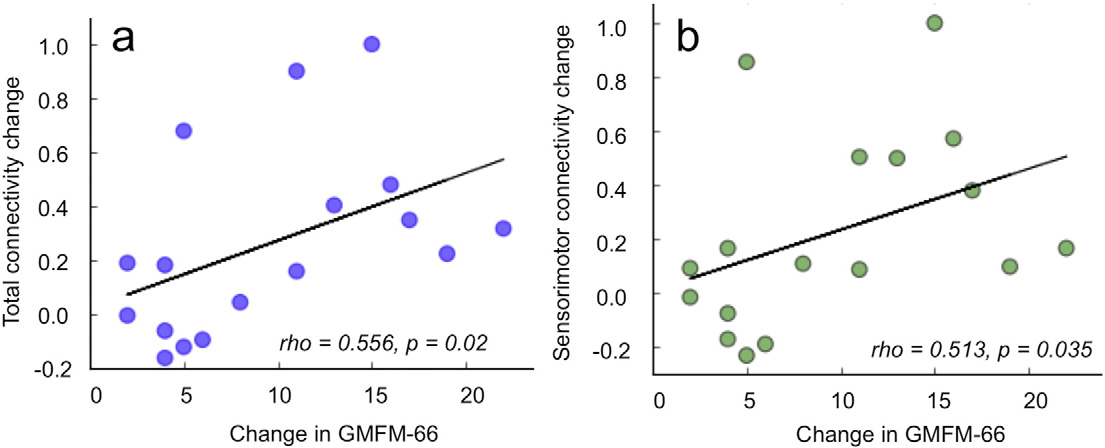
Using whole brain connectome analyses, two measures of brain connectivity change were generated, total connectivity change, reflecting the total increase in connectivity throughout the brain, and sensorimotor connectivity change, reflecting the change in connectivity specifically within the sensorimotor network. Changes in brain connectivity were examined in relation to changes in functional abilities as measured by GMFM-66 score changes. (a) Statistically significant relationships between total connectivity change and GMFM-66 score change (p = 0.020), and (b) sensorimotor connectivity change and GMFM-66 score change (p = 0.035) were observed.

Our connectome analyses revealed several key findings. First, statistically significant relationships between the total connectivity change and GMFM-66 score change (p = 0.020) (Fig. 1a), and between the sensorimotor connectivity change and GMFM-66 score change (p = 0.035) (Fig. 1b), were observed. These correlations between structural and functional changes suggest functionally relevant plasticity and WM reorganization in response to therapy. In comparison, the change in connectivity for any given node to the rest of the brain, or the change in any given edge, did not show significant correlation with functional improvement, which was expected due to the heterogeneity of symptoms in the cohort.

**Fig. 2:**
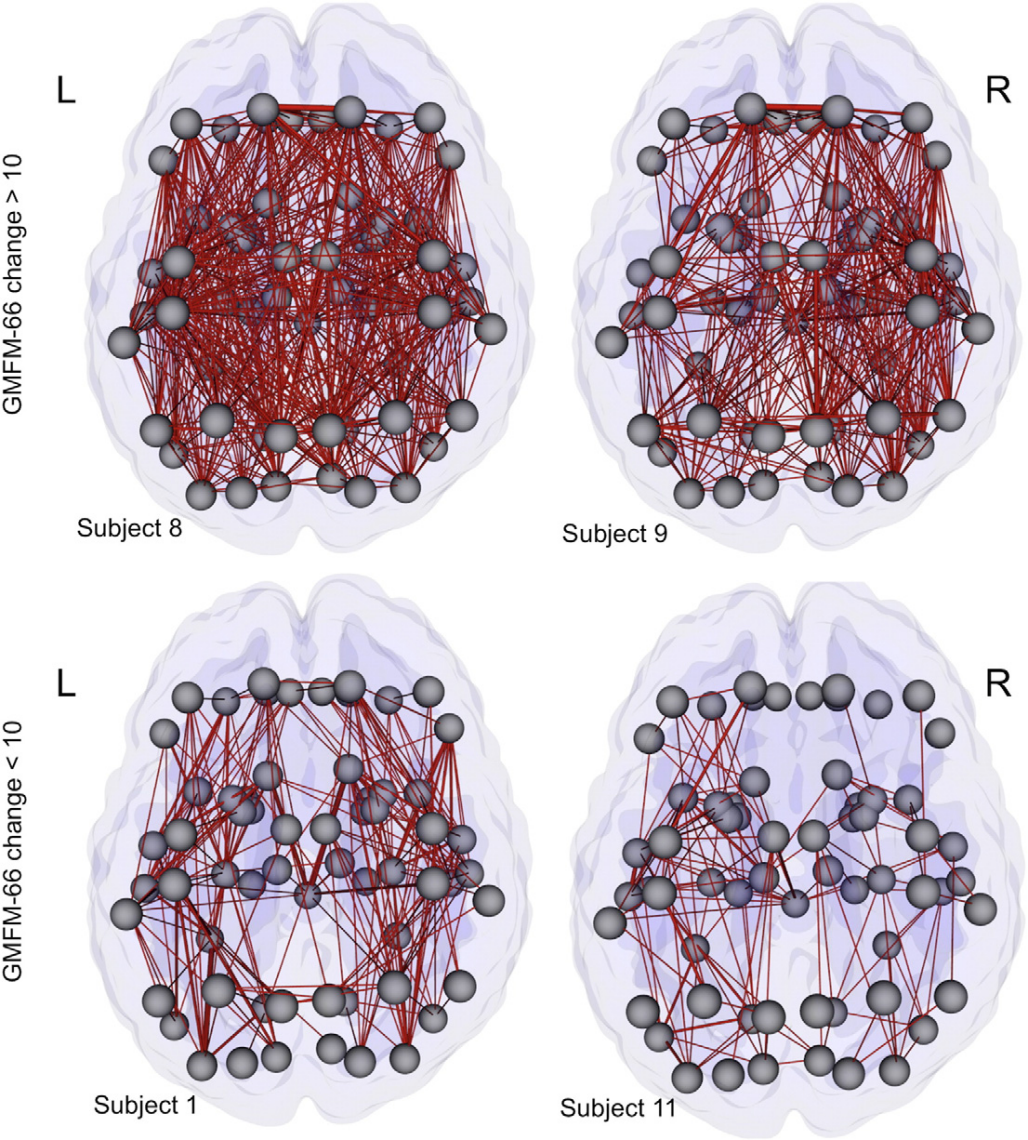
The distribution of connectivity increases over a 2-year period throughout the whole brain for four representative subjects. To generate these connectome maps, the change in each edge was normalized by the total WM volume to minimize the effect of age-related brain volume growth. The gray spheres represent the nodes, and the tubes between the nodes represent positive changes in edges over a 2-year period, with their thicknesses proportional to the magnitudes of the changes.

**Fig. 3:**
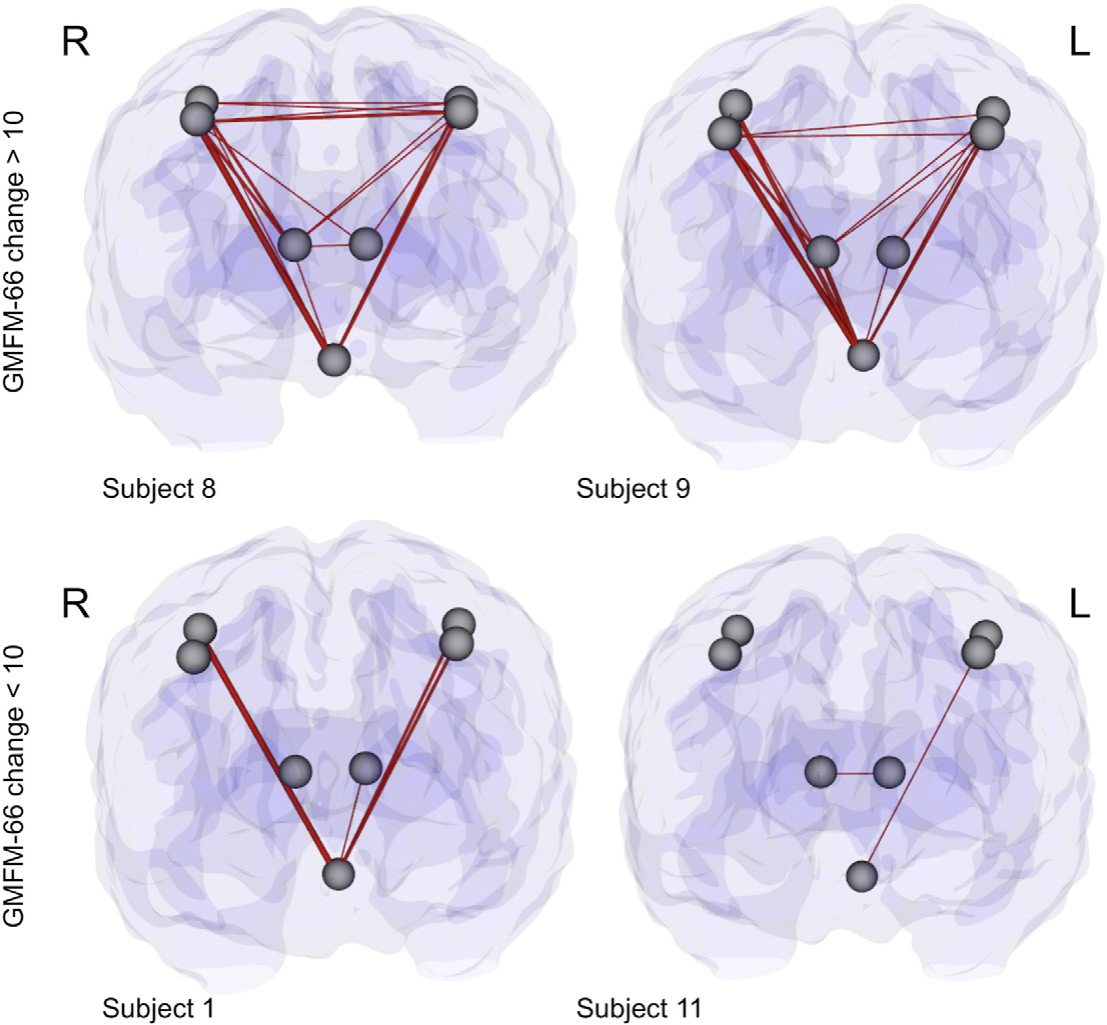
The distribution of connectivity increases over a 2-year period within the sensorimotor system for four representative subjects. To generate these connectome maps, the change in each edge was normalized by the total WM volume to minimize the effect of age-related brain volume growth. The gray spheres represent the nodes, and the tubes between the nodes represent positive changes in edges over a 2-year period, with their thicknesses proportional to the magnitudes of the changes.

For graphical illustration, connectome maps representing the 2-year connectivity changes in four representative individuals (subjects 8 and 9 for significant responders, and subjects 1 and 11 for moderate responders) are shown in Figs. 2 and 3, respectively, throughout the brain and within the sensorimotor system. In subjects with significant functional improvements (e.g. subjects 8 and 9), the numbers of edges showing increased connectivity are far greater than those in moderate responders (e.g. subjects 1 and 11). These improvements are diffusely distributed throughout the brain, and include connections that are associated with a variety of functional networks well beyond the sensorimotor system.

**Fig. 4:**
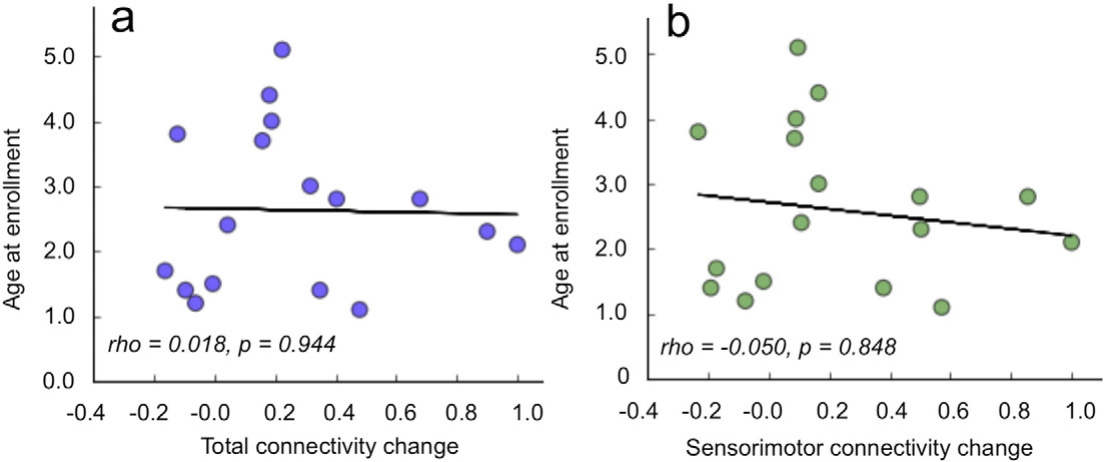
(a) The age of the child at the time of enrollment was not significantly correlated with total (p = 0.944) or (b) sensorimotor connectivity change (p = 0.848).

**Fig. 5:**
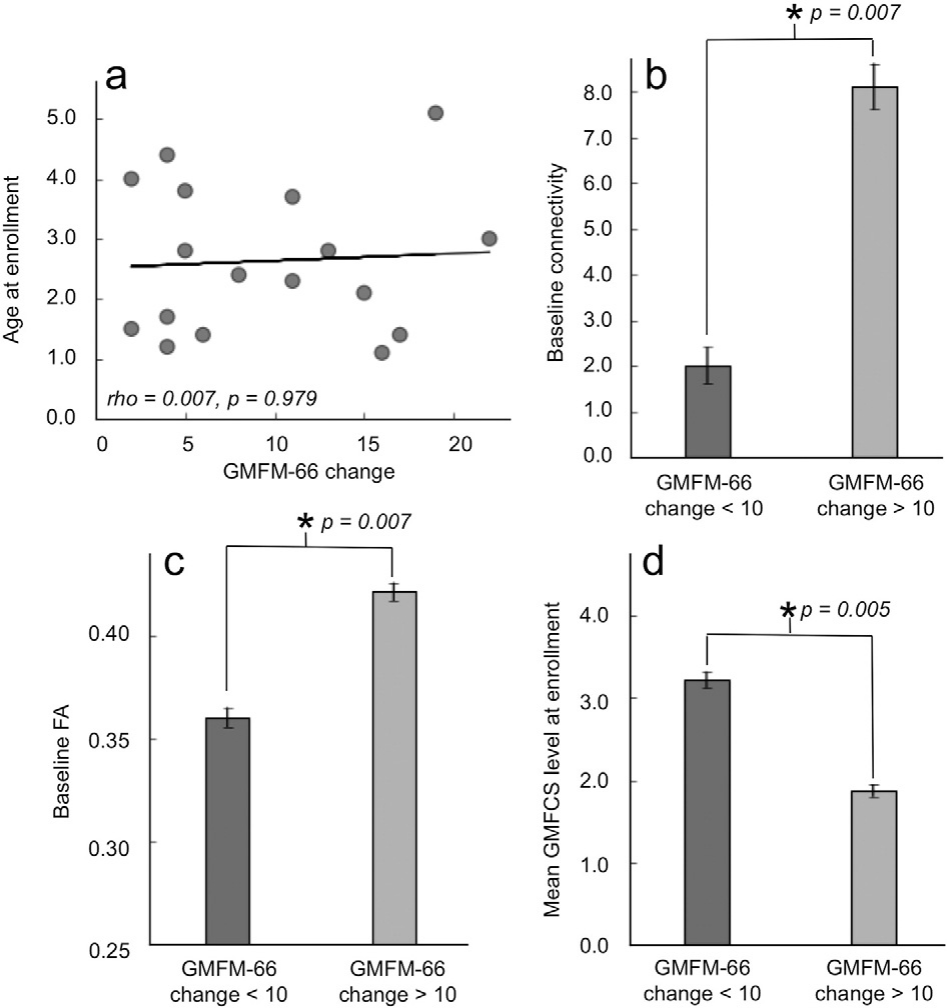
(a) No relationship between enrollment ages and GMFM-66 score changes was observed in this cohort (p = 0. 979). (b) Children with the most improved functional scores (GMFM-66 score changes > 10) demonstrated significantly higher (p = 0.007) connectivity at the time of enrollment as compared to children who showed more moderate functional improvement over the 2-year period (GMFM-66 score changes < 10). (c) Children with the most improved functional scores demonstrated significantly (p = 0.007) higher FA throughout the brain at time of enrollment than did children who showed more modest functional improvements. (d) Children who showed the greatest functional improvements were classified at lower GMFCS levels at the initial time point (indicating higher levels of gross motor function) than did children who improved more modestly. The group difference in mean GMFCS level was highly significant (p = 0.005).

It was also found that the age of the child at the time of enrollment (initial time point) was not significantly correlated with total (p = 0.944) or sensorimotor connectivity change (p = 0.848) (Fig. 4a,b). Furthermore, no relationship between enrollment ages and GMFM-66 score changes was observed in this cohort (p = 0. 979, Fig. 5a). However, children with the most improved functional scores (GMFM-66 score changes > 10) demonstrated significantly higher (p = 0.007) baseline connectivity as compared to children who showed more moderate functional improvement over the 2-year period (GMFM-66 score changes < 10) (Fig. 5b). Additionally, children with the most improved functional scores demonstrated significantly (p = 0.007) higher baseline FA than children who showed more modest functional improvement (Fig. 5c). Not surprisingly, children who showed the greatest functional improvements were classified at lower GMFCS levels at the time of enrollment (indicating higher levels of gross motor function) than did children who improved more modestly (Fig. 5d), a finding that was expected given the previously demonstrated relationship between levels of functional ability and baseline connectivity (Englander et al., 2013) and the observed relationship between baseline connectivity and functional improvement (Fig. 5b). This group difference in mean GMFCS levels was significant with p = 0.005. These findings offer possible new insight into which children are likely to most readily show the most positive outcomes following various rehabilitative therapies over a 2-year period.

## 4 Discussion

Our results reveal statistically significant relationships between brain connectivity changes and functional outcomes in children with CP. The observed changes were diffuse (not limited to the sensorimotor system), consistent with prior evidence of diffuse structural deficits in CP (Nagae et al., 2007; Englander et al., 2013). Furthermore, it was found that enrollment ages were not correlated with functional or structural outcomes, however it was demonstrated that children with higher connectivity and FA at the time of enrollment showed better functional outcomes after 2 years.

### 4.1 Relationship between brain connectivity changes and functional outcomes

The connectivity changes were associated with a variety of functional networks. In general, children who showed greater functional improvement showed more positive connectivity changes throughout the brain and in the sensorimotor system, and these changes were not associated with a specific subset of nodes.

The GMFM-66 is a measure of gross motor function that reflects more than the ability to use isolated movements, and it is designed to capture changes in ability to combine isolated movement into functional movement that requires coordination between body parts (Russell, 2002). However, the fact that changes in the GMFM-66 are correlated with diffuse changes in connectivity may reflect the fact that functional movement of the body as a whole, in coordination with the environment, may require coordinated use of the brain, rather than the isolated use of what is classically considered the sensorimotor system. Additionally, change in one aspect of the system may provide permissive conditions for the entire neural system to function more efficiently, suggesting that study of the connectivity of the entire system may be as more important, if not more important, than study of individual parts.

### 4.2 Relationship between age at time of enrollment and functional outcomes

In this study we did not observe a correlation between functional outcomes and enrollment ages. This interesting finding is contrary with the common understanding that younger brains have a greater propensity for plasticity/functional change (Kennard, 1942). It has been hypothesized that the increased potential for plasticity at young ages is due to the excess in synaptic connections that are eventually pruned by mechanisms associated with experience (Low and Cheng, 2006; Cramer et al., 2011). Furthermore, it has been shown since early CP treatment literature that early diagnosis and treatment result in better outcomes (Bobath, 1967).

There are several plausible explanations for this finding. One potentially exciting hypothesis is that the autologous cord blood transfusion is playing a role in improving functional outcomes. It is possible that the truly relevant correlation between age and eventual levels of functional improvement may be between age at the time of autologous cord-blood transfusion and GMFM-66 change scores, and not age at the time of enrollment. However, this analysis is not yet possible at this stage of our double-blind clinical trial. We expect a specific follow-up analysis to be carried out at a later time when the timing of the autologous cord blood transfusion is revealed. An alternative, though less exciting, explanation could be related to the relatively narrow age range of our cohort. The median age of this cohort was 2.4, with a maximum age of 5.1 and minimum age of 1.1 at the time of enrollment. It is possible that this age range may not have a significantly differential impact on potential for functional improvement.

### 4.3 The impact of initial functional/structural deficits at the time of enrollment on propensity for functional change

It was found that children who had relatively lower functional impairment ultimately demonstrated better functional outcomes during the course of treatment. This finding is suggestive of two neuroscience principles – Hebbian learning and long term potentiation – which indicate that synaptic communication between neuronal assemblies will, over time, lead to an increase in connection strength and efficiency between those assemblies (Bliss and Collingridge, 1993; Vidyasagar et al., 2014), which could potentially lead to structural changes in the brain. If the initial communication between neuronal assemblies does not exist, interventions aimed at facilitating plasticity and increased processing efficiency will have a more difficult time in inducing a functionally significant change, a hypothesis that is directly reflected in these findings. These data do demonstrate that children with initially higher levels of connectivity who underwent therapy were more readily able to achieve a functionally significant change to WM structure. However, this is not to say that children with lower connectivity at enrollment or higher levels of disability are not able to achieve a functionally significant change, it just suggests that achieving the same levels of improvement may require a slightly different therapeutic strategy.

Likewise, it was found that children with a higher FA at the time of enrollment demonstrated more improved GMFM-66 scores over the 2-year period. While FA values could be reflecting multiple different underlying anatomical scenarios, a higher FA is generally accepted to reflect healthier WM tracts and thus better structural integrity (Assaf and Pasternak, 2008), consistent with the previous points.

### 4.4 Relationship between treatment and structural/functional changes

Children in this cohort continued their typical course of treatment throughout the study, which included combinations of physical, developmental, and/or occupational therapies (Table 2), as well receiving autologous cord blood transfusions at either the initial or second visit. At the final time point, when functional/structural changes were assessed, all children had received a cord blood transfusion, but the researchers involved in analysis were blinded to the time point at which the experimental treatment was administered. Therefore, we are not currently able to attribute the functional changes seen in this study to the influence of the stem-cell therapy specifically, or to any other specific aspect of the individualized course of therapy. Nevertheless, and importantly, our neuroimaging data indicate that brain connectivity can serve as a sensitive biomarker of improved functional ability in children with CP undergoing treatment. Further, in future analyses, this biomarker can be used to determine the underlying mechanisms of the observed functional improvements, potentially helping to identify whether the autologous cord blood therapies are effective in facilitating better functional outcomes in these children, upon un-blinding of the timing of the transfusions at the completion of this study.

### 4.5 Technical limitations

Neuroimaging, specifically DTI, can provide specific metrics of brain microstructure non-invasively and at multiple time points in the same individual. However, there are notable limitations to the tensor model and the streamline tractography methods used here. The tensor model assumes that fiber populations are homogeneous within a voxel, and tractography algorithms based on the principal diffusion direction are unable to resolve regions of crossing white matter pathways (Mori and Zhang, 2006). These regions would benefit from a more sophisticated methodology employing high angular resolution diffusion imaging (HARDI) (Frank, 2001; Tuch et al., 2002).

## 5 Conclusions

In summary, our results demonstrate that changes in brain structural connectivity are correlated with functional improvements during therapy in children with CP. They confirm previous findings that CP symptoms are related to a diffuse network of WM deficiencies, and also suggest that functional improvements are similarly associated with widespread changes in WM organization. Our findings also indicate that children with greater structural connectedness and WM health at the time of enrollment are likely to achieve more favorable functional outcomes following therapy, at least within the 2-year time period and with the treatments in this study. Importantly, we have identified that brain connectivity change can serve as a biomarker of functional change in children with CP. Following unblinding of the timing of the administration of the autologous stem cell therapy, this biomarker can be further used to determine the mechanisms of the observed functional improvements, and potentially help to identify whether this experimental treatment is effective in facilitating better functional outcomes in these children.

## Figures and Tables

**Table 1:** Demographic information of the CP patient cohort.

Subject	Age enrollment (years)	GMFCS level at enrollment	GMFM-66 change over 2 years	Abnormal movements	Typography
1	1.7	IV	4	Spasticity with dystonia	Q
2	2.4	II	8	Spasticity	D
3	5.1	I	19	Spasticity with dystonia	H (Lh)
4	1.2	IV	4	Spasticity with dystonia	Q
5	4.0	II	2	Spasticity with dystonia	H (Lh)
6	4.4	III	4	Spasticity	D
7	2.3	II	11	Spasticity	H (Rh)
8	1.4	II	17	Spasticity	H (Lh)
9	1.1	I	16	Spasticity with dystonia	H (Rh)
10	3.7	II	11	Spasticity	H (Rh)
11	1.5	IV	2	Spasticity with dystonia	Q
12	3.8	IV	5	Spasticity with dystonia	Q
13	2.1	II	15	Spasticity	D
14	2.8	II	13	Spasticity with dystonia	H (Lh)
15	2.8	II	5	Spasticity	D
16	1.4	IV	6	Spasticity predominant, mixed with dystonia	Q
17	3.0	III	22	Spasticity predominant, mixed with spasticity	T (LUE)

Q — quadriplegic, D — diplegic, H (Lh) — hemiplegic left hemisphere, H (Rh) — hemiplegic right hemisphere, T (LUE) — tetraplegic left upper extremity.

**Table 2:** List of therapies.

Subject	Therapy (before enrollment)	Therapy (time of enrollment–year 1)	Therapy (year 1–year 2)
1	PT initiated at 0–6 months (4 hours/month), OT initiated at 0–6 months (4 hours/month), DT initiated 12–24 months (4 hours/month)	PT/OT/DT/LT (4 hours/month)	PT (8 hours/month), OT (4 hours/month), LT (8 hours/month)
2	PT initiated at 12–24 months (2 hours/month), LT initiated 24–36 months (4 hours/month)	PT (4 hours/month), OT, LT (1 hour/month), vision, hearing (0.5 hours/month)	PT (2 hours/month), OT/LT/DT/vision/hearing (1 hour/month)
3	PT initiated >3 years, OT initiated 24–36 months (4 days/week), LT initiated 24–26 months (4 days/week)	PT (2 days/week), OT (2 days/week), LT (2 days/week)	PT (1 day/week), OT (1 day/week), LT (1 day/week)
4	PT initiated 0–6 months (2 hours/month), OT initiated 0–6 months (2 hours/month), vision therapy initiated 12–24 months	OT (4 hours/month), LT (4 hours/month), Anat Baniel Method (6 hours/month)	PT (16 hours/month)
5	PT initiated 0–6 months (3 hours/month), OT initiated 0–6 months (4 hours/month), LT initiated 0–6 months (4 hours/month)	PT (3 hours/month), OT (4 hours/month), LT (2 hours/month)	OT (1 day/week), LT (1 day/week), Vision (1 day/week)
6	PT initiated 24–36 months (3 days/week), OT initiated 24–26 months (3 days/week), LT initiated 24–36 months (2 days/week)	PT/OT/LT (5 days/week)	PT/OT/LT (3 days/week)
7	PT initiated 0–6 months (5 hours/month), OT initiated 6–12 months (3 hours/month)	PT (7 hours/month), OT (5 hours/month)	PT (8 hours/month), OT (6 hours/month)
8	PT initiated 0–6 months (1 day/week), OT initiated 6–12 months (2 days/week)	PT (4 hours/month), OT (4 hours/month), LT (2 hours/month)	PT (6 hours/month), OT (6 hours/month)
9	PT initiated 6–12 months (2 hours/month), OT initiated 6–12 months (2 hours/month)	OT (4 hours/month)	None
10	PT initiated 12–24 months (1 hour/month), OT initiated 12–24 months (2 hours/month), LT initiated 24–36 months (2 hours/month)	PT (4 hours/month), OT (4 hours/month), LT (6 hours/month)	PT (5 hours/month), OT (2 hours/month), LT (4 hours/month)
11	PT initiated 6–12 months (8 hours/month), OT initiated 6–12 months (8 hours/month)	PT (4 hours/month), OT (4 hours/month), LT (8 hours/month), vision therapy (8 hours/month), infant school (8 hours/month)	PT (8 hours/month), OT (8 hours/month), LT (3 hours/month)
12	PT initiated 0–6 months (4 hours/month), OT initiated 0–6 months (4 hours/month), LT initiated 6–12 months (2 hours/month), DT initiated 12–24 months, Vision therapy initiated 12–24 months	PT (4 hours/month), OT (4 hours/month), LT (4 hours/month)	PT (6 hours/month), OT (6 hours/month), LT (4 hours/month)
13	PT initiated 12–24 months, (2 hours/month), OT initiated 12–24 months (2 hours/month), LT initiated 12–24 months	PT (1 hour/month), OT (1 hour/month)	PT (2 hours/month)
14	PT initiated 12–24 months (4 hours/month), OT initiated 12–24 months (4 hours/month)	PT (4 hours/month), OT (4 hours/month)	PT (4 hours/month), OT (4 hours/month), Hippotherapy (4 hours/month)
15	PT initiated 6–12 months (8 hours/month), OT initiated 12–24 months (4 hours/month), LT initiated 12–24 months, Vision therapy initiated 12–24 months, Feeding therapy initiated 12–24 months (4 hours/month)	PT (8 hours/month), OT (4 hours/month)	PT (4 hours/month)
16	PT initiated 0–6 months (12 hours/month), OT initiated 6–12 months (12 hours/month), DT initiated 6–12 months (4 hours/month), Feeding therapy initiated 6–12 months (4 hours/month)	PT (12 hours/month), OT (12 hours/month), LT (6 hours/month), DT (4 hours/month), Hippotherapy (4 hours/month)	PT (10 hours/month), OT (10 hours/month), LT (8 hours/month), Hippotherapy (4 hours/month)
17	PT initiated 12–24 months (8 hours/month), OT initiated 12–24 months (4 hours/month)	PT (12 hours/month), OT (4 hours/month), Hippotherapy (2 hours/month)	PT (12 hours/month), OT (4 hours/month), Hippotherapy (2 hours/month)
